# Chikungunya Virus: An Emergent Arbovirus to the South American Continent and a Continuous Threat to the World

**DOI:** 10.3389/fmicb.2020.01297

**Published:** 2020-06-26

**Authors:** Marcela S. Cunha, Pedro A. G. Costa, Isadora Alonso Correa, Marcos R. M. de Souza, Pedro Teles Calil, Gustavo P. Duarte da Silva, Sara Mesquita Costa, Vinícius Wakoff P. Fonseca, Luciana J. da Costa

**Affiliations:** Departamento de Virologia, Instituto de Microbiologia Paulo de Góes, Universidade Federal do Rio de Janeiro, Rio de Janeiro, Brazil

**Keywords:** Chikungunya virus, emergent arbovirus, virus–cell interaction, pathogenesis, epidemiology

## Abstract

Chikungunya virus (CHIKV) is an arthropod-borne virus (arbovirus) of epidemic concern, transmitted by *Aedes* ssp. mosquitoes, and is the etiologic agent of a febrile and incapacitating arthritogenic illness responsible for millions of human cases worldwide. After major outbreaks starting in 2004, CHIKV spread to subtropical areas and western hemisphere coming from sub-Saharan Africa, South East Asia, and the Indian subcontinent. Even though CHIKV disease is self-limiting and non-lethal, more than 30% of the infected individuals will develop chronic disease with persistent severe joint pain, tenosynovitis, and incapacitating polyarthralgia that can last for months to years, negatively impacting an individual’s quality of life and socioeconomic productivity. The lack of specific drugs or licensed vaccines to treat or prevent CHIKV disease associated with the global presence of the mosquito vector in tropical and temperate areas, representing a possibility for CHIKV to continually spread to different territories, make this virus an agent of public health burden. In South America, where Dengue virus is endemic and Zika virus was recently introduced, the impact of the expansion of CHIKV infections, and co-infection with other arboviruses, still needs to be estimated. In Brazil, the recent spread of the East/Central/South Africa (ECSA) and Asian genotypes of CHIKV was accompanied by a high morbidity rate and acute cases of abnormal disease presentation and severe neuropathies, which is an atypical outcome for this infection. In this review, we will discuss what is currently known about CHIKV epidemics, clinical manifestations of the human disease, the basic concepts and recent findings in the mechanisms underlying virus-host interaction, and CHIKV-induced chronic disease for both *in vitro* and *in vivo* models of infection. We aim to stimulate scientific debate on how the characterization of replication, host-cell interactions, and the pathogenic potential of the new epidemic viral strains can contribute as potential developments in the virology field and shed light on strategies for disease control.

## Introduction

The Chikungunya virus (CHIKV) is an arthropod-borne virus (arbovirus) globally distributed to the tropical areas that has recently spread to subtropical areas and the western hemisphere. CHIKV is an arthritogenic virus belonging to the family *Togaviridae*, genus *Alphavirus*, and is the etiological agent of the acute febrile illness Chikungunya fever (CHIKF) that caused millions of human cases since major outbreaks starting in 2004 (Sharp et al., 2014). This disease was named after the Makonde (Kimakonde) language from the south of Tanzania, which means “to bend over,” referring to the posture assumed by individuals that display the most severe forms of the disease with extreme and incapacitating joint pain. Although CHIKV infection is associated with low mortality rates, it imposes severe morbidity to the acute-infected individuals. The debilitating joint pain can persist for several months to years as a clinical outcome known as “post-chikungunya chronic polyarthralgia” (pCHIKV-CPA), which deeply affects the patient’s quality of life (Consuegra-Rodríguez et al., 2018). Since 2004, substantial urban outbreaks of CHIKV infection have occurred throughout the tropical and subtropical regions of the world, particularly in geographical areas inhabited by the vectors *Aedes* spp. mosquitoes (Petersen and Powers, 2016). More recently, CHIKV outbreaks occurred in Africa, Asia, Europe, the Americas, and the Pacific islands (Petersen and Powers, 2016). This unprecedented spread of CHIKV infections was accompanied by high morbidity, several cases of neuropathies, and atypical disease presentations, making CHIKV a major global health threat. Facing this scenario, the characterization of the infectious and pathogenic potential of the actual circulating virus isolates will help to understand and, more effectively, control the disease.

The first isolation of CHIKV, and the report of an epidemic, occurred in 1952/53 in Tanganyika Province, actual Tanzania, with the infected individual presenting disabling joint pains, severe fever, and eventually rash (Lumsden, 1955; Ross, 1956). The bite of infected female mosquitoes transmits the virus, and its circulation could be related to two different cycles of transmission: (1) a sylvatic cycle where enzootic transmissions between non-human primates and *Aedes* spp. mosquitoes, such as *Ae.* (Diceromyia) *furcifer*, *Ae.* (Diceromyia) *taylori, Ae*. (Stegomyia) *luteocephalus*, *Ae.* (Stegomyia) *africanus*, and *Ae.* (Stegomyia) *neoafricanus*, which occasionally spilled over to humans; (2) an urban cycle where humans and *Ae. aegypti* and *Ae. albopictus* are involved. The importance of the sylvatic cycle could be highlighted in a recent study that detected the virus in non-human primates from Malaysia and revealed a high similarity between human and non-human primate sequences of CHIKV. Thus, these monkeys maybe both hosts and reservoirs for CHIKV (Suhana et al., 2019). In addition, CHIKV has been detected in other zoophilic mosquitoes *Ae. dalzieli*, *Ae. argenteopunctatus*, *Cx. ethiopicus*, and *An. rufipes* suggesting that other species may participate in a secondary sylvatic cycle (Diallo et al., 1999).

**FIGURE 1:**
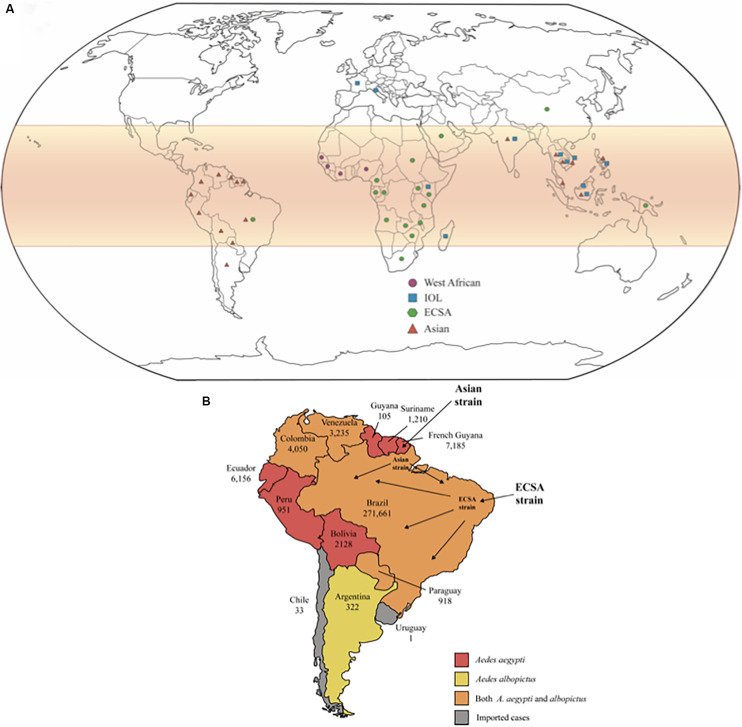
**(A)** Global distribution of CHIKV lineages. CHIKV infections are more likely to occur in tropical and sub-tropical regions of the globe, highlighted in red on the map. The geometric forms represent the different lineages of CHIKV that are currently in circulation. **(B)** The number of confirmed cases is shown for each country individually. There is not autochthonous transmission reported in Chile and Uruguay, only imported cases. The Asian strain first reached South America by French Guyana, but ECSA strain has arrived by northeast Brazil and got predominated in Brazil. The colors represent the circulation of Aedes aegypti and albopictus in each country, as indicated in the subtitle.

Phylogenetic studies show that CHIKV originated from Africa, although the specific region where the virus evolved could not be pinpointed, and subsequently spread to Asia. These studies also classify viral isolates into three main lineages: the enzootics East/Central/South African (ECSA), West African, and the endemic/epidemic Asian strains. The Asian lineage could be sub-divided into two clades: the Indian clade, which was extinct, and the Southeast Asian lineage that continues to circulate (Powers et al., 2000; Volk et al., 2010). The recent epidemic that affected La Réunion Island and other islands from Indian Ocean revealed a new strain derived from the ECSA group, which was named the India Ocean lineage (IOL) (Njenga et al., 2008). The distribution of CHIKV genotypes worldwide is represented in Figure 1A.

Mutations in the viral genome impact at viral propagation and adaptation of these lineages in different vectors. *Ae. aegypti* and *Ae. albopictus* mosquitoes are the main vectors in the urban cycle of CHIKV transmission. Studies showed that genomic differences amongst circulating CHIKV accounted for its transmission from each of these vectors. For instance, the presence of the A226V variant on the envelope (E1) gene of CHIKV was related to an increase in viral infectivity, dissemination, and transmission in *Ae. albopictus*, resulting in the wide spread of the virus (Tsetsarkin et al., 2007). This mutation did not confer any advantage to transmission in *Ae. aegypti*. Followed by the selection of A226V, adaptation substitutions L210Q and K252Q (E2 protein) that arose independently in the IOL strain in India are associated with a greater increase of CHIKV dissemination in *Ae. albobictus* vector (Tsetsarkin and Weaver, 2011; Tsetsarkin et al., 2014). Still, variants K211E, in the E1 gene, and V264A, in the envelope (E2) gene, lead to an increase in viral dissemination and transmission for *Ae. aegypti* but not for *Ae. Albopictus* (Agarwal et al., 2016). Moreover, the T98A variant in E1 enhances the vector-adaptability effect of A226V, since epistatic interactions between E1-98T and E1-A226V are restrictive (Tsetsarkin et al., 2011). In another study, variant G60D in the E2 increased CHIKV infectivity in *Ae. albopictus* in the presence of either alanine or valine at position 226 in E1 protein. This change also increases infectivity in *Ae. aegypti.* The E2 variant, I211T, increases the CHIKV infectivity exclusively for *Ae. albopictus* but only when associated with A226V change. I211T variant could be related to the maintenance of CHIKV in the enzootic Africa cycle since it was detected in most sequences from the ECSA clade obtained before 2005 (Tsetsarkin et al., 2009). Mutations occurring at the 3′-UTR could also contribute to vector adaptability, since a 177 nt duplication found in the Caribbean strain of CHIKV and confirmed in sequences from Mexico, Trinidad, and the Dominican Republic, conferred a growth advantage in insect cell cultures to viruses harboring this duplication over the Asian strain and other Caribbean strains lacking the duplication (Stapleford et al., 2016). The most relevant variants, as well as their impact on each vector and in virus infectivity, are summarized in Table 1.

**TABLE 1:** CHIKV variants associated with vector adaptability.

Variant	Gene	Function	References
A226V	E1	Increased infectivity, transmission, and dissemination in *Ae. Albopictus*	Schuffenecker et al., 2006; Tsetsarkin et al., 2007
T98A	E1	Enhanced vector adaptability of A226V	Tsetsarkin et al., 2011
K211E	E1	Epistatic effect – Increased dissemination in *Ae. Aegypti*	Agarwal et al., 2016
V264A	E2		
L210Q	E2	Enhanced disseminated infection in *Ae. albopictus* and fitness increment of A226V variant	Tsetsarkin and Weaver, 2011; Tsetsarkin et al., 2014
K252Q	E2	Enhanced disseminated infection in *Ae. albopictus* and fitness increment of A226V variant	Tsetsarkin and Weaver, 2011; Tsetsarkin et al., 2014
I211T	E2	Increased infectivity in *Ae. albopictus* when associated with A226V	Tsetsarkin et al., 2009
G60D	E2	Increased infectivity in *Ae. aegypti* and *albopictus*	Tsetsarkin et al., 2009
177 nt insertion	3′ UTR	Increased viral replication in insect cell culture	Stapleford et al., 2016

Mainly during the 1960s and 1970, epidemics of CHIKV were restricted to Africa and Southeast Asia, in countries like South Africa, Democratic Republic of Congo, Uganda, Indonesia, Thailand, and India. However, this scenario started to change in 2004 with reports of an outbreak in Lamu, Kenya, beginning in May and reaching its peak in July, with an estimated 75% of the island’s population affected (Sergon et al., 2008). The disease then spread through Mombasa and the Comoros islands. Other islands from the Indian Ocean were affected, including La Réunion Islands where, between March 2005 and April 2016, 244,000 cases were reported (Renault et al., 2007).

The variant E1-A226V on the viral envelope glycoprotein was detected for the first time in viruses that circulated during the La Reunion epidemic (Tsetsarkin and Weaver, 2011). This adaptation of CHIKV to *Ae. albopictus* allowed that regions of the planet such as Italy (during July and August 2007) (Fadila and Failloux, 2006; Rezza et al., 2007) and France (during 2010 and 2014) (Grandadam et al., 2011; Delisle et al., 2015), that never had reported CHIKF cases, experienced the occurrence of CHIKV disease.

The CHIKV adaptation to *Aedes albopictus* has constantly been associated to spread of CHIKF to new areas of the globe. In fact, full-length viral sequences unraveled unique adaptive variants in, at least, three occasions, that conferred selective advantage for CHIKV transmission by *Ae. albopictus* (Tsetsarkin et al., 2007; Beesoon et al., 2008; De Lamballerie et al., 2008; Dubrulle et al., 2009; Severini et al., 2018).

## Epidemiology of CHIKV on the South American Continent

In 2013, the American continent reported the first cases of autochthonous transmission of CHIKV on the island of Saint Martin and Martinique Islands. In January 2014, CHIKV transmissions occurred in several Caribbean islands, including Dominica, Anguilla, British Virgin Islands, Saint Barthelemy, Guadeloupe, the Federation of St. Kitts and Nevis, Dominican Republic; and Saint Vincent and the Grenadines. The number of infected people overcame 30,000 cases in 4 months. It is interesting to note that at these sites, only *Ae. aegypti* mosquitoes were the circulating vectors (Nasci, 2014; Leparc-Goffart et al., 2014), indicating that different from the explosion of CHIKV infections in La Reunion related to viral adaptation to another mosquitoes vector, other factors contributed to the introduction and spread of CHIKV in the American continent. Lanciotti and Valadere demonstrated that the strain of CHIKV circulating in the Caribbean Islands belongs to Asian genotype and is closely related to strains circulating in Indonesia (2007), China (2012), Yap Islands (2013), and Philippines (2013) (Lanciotti and Valadere, 2014).

In February 2014, CHIKV had already reached continental territory, when autochthonous infections were observed in French Guiana, the first country in South America to declare CHIKV infection. At this point, the dispersion of CHIKV to other American countries was only a matter of time. From 2014 to 2015, more than 16,000 individuals were infected in French Guiana. Importantly, infections presented several atypical cases, such as neurological disorders, cardio-respiratory failure, acute hepatitis, acute pancreatitis, renal disorders, and muscular impairment. Only two deaths associated with CHIKF during this period were documented (Bonifay et al., 2018; Figure 1B).

CHIKV cases arose in Venezuela in June 2014, from recent travelers from the Dominican Republic or Haiti, and in July 2014, autochthonous transmissions were reported. Phylogenetic analysis showed that the CHIKV circulating in Venezuela clustered to the Asian genotype (Caribbean clade) and did not harbor the main substitutions associated with *Ae. albopictus* viral adaptation (Camacho et al., 2017).

Ecuador was another country that early confirmed community transmission of CHIKV. Berry et al. (2020) showed that CHIKV was introduced into Ecuador at multiple time points in 2013–2014, and these introductions were all associated with the Caribbean islands, despite the increasing influx of Venezuelan citizens. From 2014 to 2017, Ecuador reported 35,714 CHIKF cases. The transmission for two or more years after the 2015 epidemic peak suggests that CHIKV has become endemic in this country. The CHIKF outbreaks in Ecuador were associated with the Asian strain which harbors the E1:A98T and E1:K211E amino acid changes. Since *Ae. aegypti* is the main mosquito vector in Ecuador this data indicates that CHIKV had not acquired all the adaptative substitutions necessary to increase viral fitness within this vector (Berry et al., 2020).

CHIKV autochthonous cases were confirmed in Colombia in September 2014, and during the epidemics (2014–2015) more than 460,000 cases diagnosed of CHIKF by clinical features were reported, with the majority of them occurring in women, with 12 fatal cases reported. The rate of new infections is decreasing over time, although Colombia is the country with the third-highest number of infections, according to the Pan American Health Organization (PAHO). The characterization of Colombian CHIKV genomes determined that it belongs to the Asian strain and clustered with three distinct Asian strain branches: Panama (Caribbean Colombia, Huila); Nicaragua (Cauca and Risaralda); and St. Barts (Bogotá, D.C), which may be the result of three independent introductions. Each subclade showed non-synonymous mutations (nsP2-A153V, Y543H, G720A; nsP3-L458P; and Capsid R78Q), and that may impact on CHIKV fitness and pathogenesis (Rico-Mendoza et al., 2019; Villero-Wolf et al., 2019; Figure 1B).

Records of CHIKV infection cases in Bolivia are extremely scarce. However, CHIKV circulated in this country since March 2015, when 204 cases were reported (Carbajo and Vezzani, 2015). In 2017, 3,367 cases were reported across the country (including clinically diagnosed only) (Escalera-Antezana et al., 2018; Figure 1B).

Since 2014, Peru has reported 27 confirmed cases of CHIKV, all of them imported from neighboring countries such as Venezuela and Colombia (Ministerio de Salud, Dirección General de Epidemiología, 2015). This country has the circulation of *Aedes aegypti* vector in 18 territories and co-circulation of other arboviruses such and ZIKV and Dengue. The first case of autochthonous transmission of CHIKV was reported in 2015 and since then, 951 cases of autochthonous transmission were confirmed in the country according to PAHO. Different regions of Peru present divergent rates of CHIKV infection, varying from 4.6 to 9.4% of all cases of febrile illness (Alva-Urcia et al., 2017; Sánchez-Carbonel et al., 2018), demonstrating that several factors could impact on the epidemiology of CHIKV infection, including the molecular diagnostics, which, in addition to being poorly established and accessible in the country, and the environmental factors, such as natural climatic events, that can increase the frequency of infections.

Some South American countries situated mostly under the Tropic of Capricorn present temperate climate, with warm summers and low temperatures in the winter season, which impair the establishment of a considerable mosquito’s population and, consequently, the transmission of arboviruses is negatively impacted. The first CHIKV imported case in Chile was described in 2014, from the Dominican Republic. Since then, all cases reported in Chile were imported, mainly from travelers returning from the Caribbean islands. Argentina, however, presented autochthonous CHIKV transmissions in 2016, and more than 320 lab-confirmed cases were reported, according to PAHO (Perret et al., 2018; Figure 1B).

In 2017, 123,087 autochthonous cases were confirmed in the American continent (Pan American Health Organization, 2020). In Brazil, unprecedented dissemination of CHIKV infections has been occurring since 2015, with an accumulated of 712,990 confirmed cases notified over a 4-year period. This outbreak had its major incidence in the Southeast and Northeast regions of the Brazilian territory, corresponding to two-thirds of all confirmed Brazilian cases mainly in periurban and highly populated urban areas of the country.

The first local transmission of the CHIKV in Brazil that occurred in September 2014, at the city of Oiapoque, state of Amapá, localized in the Northern region of Brazil was related to the Asian lineage. Soon after this first autochthonous detection, CHIKV infections from the ECSA genotype were notified in the city of Feira de Santana, Bahia state, the north-eastern region of Brazil. Asian and ECSA genotypes co-circulate in the North and Northeast regions of Brazil (Nunes et al., 2015). However, CHIKV ECSA strain spread to other northeastern states, such as Paraíba, Sergipe, Pernambuco, and Alagoas. In 2017 this strain reached the Amazon region. Interestingly, while the north and southeast regions of Brazil had the majority of CHIKV cases in 2016, Roraima, for instance, the northernmost state of Brazil located in the Amazon basin, only had its exponential increase of cases in 2017. All strains analyzed from this outbreak in Roraima were of the ESCA strain. An extended analysis demonstrated that most cases circulating in Roraima and Amapa since 2015 were of the CHIKV ECSA origin (Naveca et al., 2019). The CHIKV Asian strain was first identified in Roraima in 2014, representing people returning from Venezuela, but the infection did not spread from these two cases. This data demonstrates the high potential of CHIKV ECSA spread in the Brazilian territory.

CHIKV ECSA also reached the southeast region of Brazil, causing large outbreaks. Increasing evidence indicates that the ECSA genotype has predominated in the Southern region, especially in Rio de Janeiro. Xavier et al. (2019) sequenced 11 near-complete CHIKV genomes from clinical samples of patients from Rio de Janeiro, and together with the whole sequencing of 2 CHIKV genomes from positive individuals by Cunha et al. (2017), during the 2016 outbreak, and 10 partially sequenced samples (CHIKV E1 gene) by Souza et al. (2017), the phylogenetic reconstructions confirmed that in Rio de Janeiro the ECSA strain is the driving force of the epidemics (Figure 1B).

Phylogenetic analysis also demonstrated that the origin of ECSA strain in Rio de Janeiro was from the north-eastern region of Brazil. Xavier et al. (2019) also showed that there is high human mobility between the two regions and the epidemic waves from the north-eastern region and Rio de Janeiro state had synchronicity during late 2015 to the early months of 2016. Moreover, they estimated that CHIKV was circulating unnoticed for at least 5 months before the first reports of autochthonous transmissions in Rio de Janeiro (Xavier et al., 2019). Another work has estimated an even earlier ECSA genotype introduction in the Rio de Janeiro state. The time-scaled phylogenetic tree estimated the introduction as early as 2014 (Souza et al., 2019).

Corroborating data from Cunha et al. (2017), the genomes of the CHIVK circulating ECSA strain did not carry the E1-A226V and E2-L210Q *Ae. albopictus* adaptive changes. In fact, in Brazil, *Ae. aegypti* is the main circulating mosquito strain (Cunha et al., 2017; Souza et al., 2019; Xavier et al., 2019). Thus, it is expected that mutations that confer high viral fitness in *Ae. albopictus* have not been fixed at these locals.

Although the Brazilian ECSA CHIKV did not harbor the E1-A226V and E2-(L210Q, V264A), which were also related to CHIKV-vector adaptability (Tsetsarkin and Weaver, 2011), unique mutations such as E1-K211T, E1-N335D, E1-A377V, and E1-M407L are present together with E2-A103T (Cunha et al., 2017; Souza et al., 2017). The impact of these mutations on CHIKV adaptability to *Aedes ssp.* vectors still needs to be addressed, but as for the polymorphic E1-211K, the E1-K211E mutation has been implicated in better viral transmission for *Ae. aegypti* but not for *Ae. albopictus* (Agarwal et al., 2016). Importantly, the unprecedented spread of the ECSA strain in Brazil, which substituted the Asian strain in the north part of the country, suggests a greater potential of transmission of this strain.

The dynamics of CHIKV disease in South America, its spread, and the outcome expected can be influenced by several complex factors. The climate patterns, like pluviosity, humidity, ocean-atmosphere climate phenomenon, such as El Niño–Southern Oscillation (ENSO), as well as other parameters, as vector habitat availability, adaptability of the virus into a new vector species, cocirculation of other arboviruses, heterogeneity of health systems in each country, country’s economy and the Human Development Index, mobility of individuals (by traveling, exodus, among other reasons), the efficiency in combating disease vectors, the capacity of surveillance and epidemiological vigilance, with the proper actions to stop the outbreaks. All the previous parameters are related to viral vector biology and adaptability. In any case, the biological behavior of each CHIKV strain cannot be ruled out and the characterization of different CHIKV strains in terms of replication, virus-cell interaction, and pathogenesis urge to be determined.

### Virus Particle, Genomic Structure, and the Replication Cycle

The CHIKV viral particle carries the 11.8 Kb, single-stranded positive genomic RNA, which is arranged in two modules: the 5′ two-thirds codes for the non-structural protein (nsPs1-4) and the 3′ one-third codes for the structural proteins (CP, E3, E2, 6K, E1) (Knipe et al., 2001); additionally the 3′ one-third can be translated as a truncated polyprotein composed of CP, E3, E2, C-terminal 6K fused with a Transframe or TF peptide (Firth et al., 2008; Snyder et al., 2013). The 5′ terminus is capped with a 7-methylguanosine and the 3′ terminus is polyadenylated. The genomic RNA is enclosed by a capsid formed by 240 copies of a single Capsid (CP) protein arranged as icosahedrons with T4 symmetry. This nucleocapsid is delimited by the external phospholipid envelope formed essentially by cholesterol and sphingolipid derived from the host cell plasma membrane containing the virus glycoproteins E1 and E2. Each CP interacts with the cytosolic domain of E2. The glycoproteins are arranged as trimeric spikes composed of heterodimers of E1 and E2, and each viral particle contains 80 spikes which lead to the incorporation of 240 copies of E1 and E2 (reviewed in Knipe et al., 2001; Jin and Simmons, 2019). Glycoproteins E1 and E2 mediate CHIKV infection of susceptible cells, where E2 is responsible for receptor binding while E1 plays a role in viral-host membranes fusion.

Until recently, the cellular receptor used by CHIKV, and other arthritogenic alphaviruses, was not known, but several pieces of evidence pointed out to CHIKV use of glycosaminoglycans (Smit et al., 2002; Gardner et al., 2014; Weber et al., 2017; and reviewed in Solignat et al., 2009), T-cell immunoglobulin and mucin 1 (TIM-1) (Moller-Tank et al., 2013), and other PtdSer-binding proteins, such as Axl and TIM-4 (Jemielity et al., 2013) and prohibitin (Wintachai et al., 2012) as adsorption factors. However, Zhang et al. (2018) demonstrated that CHIKV and other arthritogenic alphaviruses, such as Ross River Virus (RRV) and Mayaro Virus (MAYV), use Mxra8 (also known as DICAM, ASP, or Limitrin) as a cell receptor for virus entry. Mxra8 is an adhesion molecule of epithelial, myeloid, and mesenchymal cells with homology to the junctional adhesion molecule that serves as the receptor for reoviruses. The immunoglobulin domains A and B of CHIKV E2 bind to Mxra8 and this binding was necessary for CHIKV mouse infection. Interestingly, infection with the CHIKV ECSA strain La Réunion did not show any requirement to use Mxra8 for viral entry, which indicates that other unknown molecules can function as CHIKV receptors. In addition, this observation demonstrates that different genotypes of CHIKV can adapt differently to the host, thus possibly indicating divergent outcomes of CHIKV disease.

Even though several studies pointed out that E2 acts on CHIKV binding to surface cell receptors, while E1 is the main protein factor involved in the intracellular process of virus entry, there is evidence that points to shared participation of the two proteins at the viral entry and its subsequent events. First, like other alphaviruses, CHIKV can use endocytosis to enter a cell, in a pH-dependent process in clathrin-coated vesicles via receptor-mediated interaction (DeTulleo and Kirchhausen, 1998; Smith and Helenius, 2004; Kielian et al., 2010). In this scenario, after CHIKV enter cells via receptor-mediated endocytosis, the acidic endosomal environment results in glycoproteins irreversible conformational changes followed by E2-E1 heterodimers dissociation and E1 rearrangement into fusogenic homotrimers that induce fusion of viral and endosomal membrane, allowing the release of the nucleocapsid into the cytosol (Voss et al., 2010). But the *Old-World Alphavirus* title (Weaver et al., 1994) makes something very clear about CHIKV: the virus, its vectors, and its final hosts have been coevolving for a long time. Therefore, other pathways did not take long to be elucidated, like the clathrin-independent, epidermal growth factor receptor substrate15 (Eps15)-dependent pathway (Bernard et al., 2010), which also takes the virus particle into the endosome. A third pathway exploited by the virus to get into an acidic cell compartment is the macropinocytosis, recently attributed to CHIKV (Lee et al., 2019), but an already well-established mechanism for other enveloped viruses, such as Ebola virus (EBOV), and non-enveloped viruses, such as adenoviruses; the Rab GTPases- and phosphoinositide-dependent maturation of the macropinosome induces its fusion to endosomal compartments (Egami et al., 2014). The low pH of acid milieu creates the proper microenvironment required to induce conformational changes in the viral envelope, dissociating E1-E2 heterodimers and forming E1 homotrimers, allowing CHIKV fusion to the endosome membrane and the release of the nucleocapsid into the target cell’s cytosol where, as it was demonstrated to the Sindbis Virus (SINV), the uncoating of the viral genomic RNA is carried out by the association of the CP and the ribosomes (Singh and Helenius, 1992).

Like other togaviruses and due to the particular arrangement of alphavirus genomic RNA, following uncoating, the CHIKV non-structural (ns) proteins are translated as polyproteins P123 and P1234, with 1,857 amino acids and 2,475 amino acids, respectively. A well-conserved opal (UGA) stop codon is present at the C-terminus of nsP3 and determines the translation of P123, which contains the nsP1, nsP2 and, nsP3 proteins. The readthrough of the opal stop codon leads to the translation of the full-length P1234, that contains the nsP4 protein, the viral RNA-dependent RNA polymerase (RdRp), in addition to the nsP1-nsP3 proteins. The readthrough frequency of the opal stop codon, determined for the SINV, is about 5–20% of the genomic mRNA translation. Therefore, the stoichiometric concentration of nsP4 is 1/20 to 1/5 of the other non-structural proteins (Shirako and Strauss, 1994).

Interestingly, some isolates of alphaviruses code an amino acid residue at the place of the opal stop codon. For instance, a SINV isolate presenting severe morbidity and mortality in mice codes for cysteine at the opal stop codon position (Suthar et al., 2005), while in ONNV both arginine and the opal stop codon are present, and a viral fitness advantage and higher infectivity in the *Anopheles gambiae* mosquito vector is related to the presence of the opal stop codon (Myles et al., 2006). Analyses by deep-sequencing of a Caribbean isolate of CHIKV (ECSA-derived IOL linage) demonstrated the presence of both the opal stop codon and arginine at the end of nsP3 coding region. The moderate disease was observed in mice infected with a Sri Lanka CHIKV isolate harboring an opal stop codon to arginine change. Sri Lanka isolate shares high similarity with the Caribbean isolate, and the opal stop codon to arginine change did not alter viral replication kinetics (Jones et al., 2017). Collectively, these data suggest that the identification of viral determinants will contribute to a better understanding of CHIKV disease severity and prognostics, and the epidemic potential of different viral strains.

**FIGURE 2:**
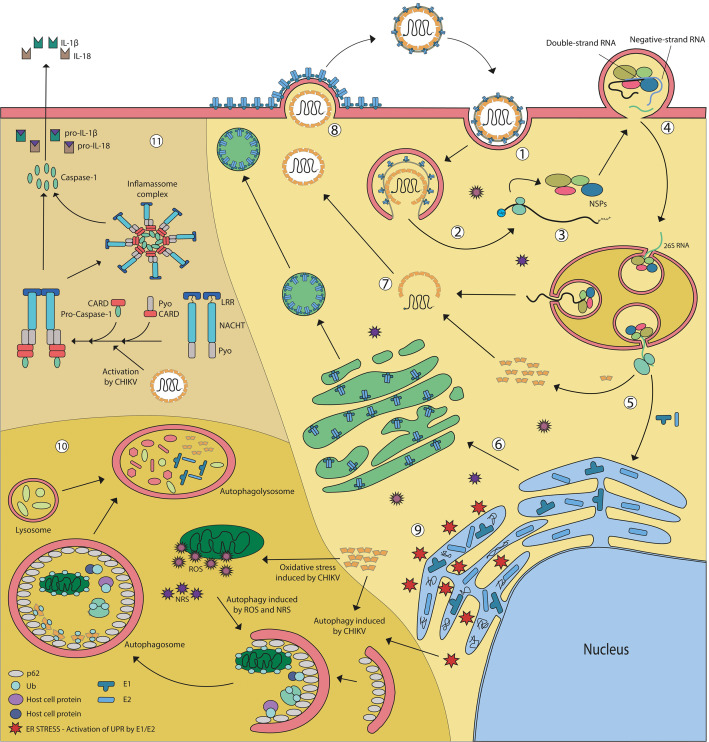
CHIKV life cycle in mammalian infected cells. **(1)** CHIKV cell binding occurs through the interaction of virus E2 protein and a still unknown cellular receptor. Like other alphaviruses, it can enter the cell by clathrin-dependent and independent endocytosis. **(2)** Once inside the endosome, the acidic environment leads to conformational rearrangement of glycoproteins followed by dissociation of E2-E1 heterodimers and E1 rearrangement into fusogenic homotrimers that induce fusion of viral and endosomal membrane, allowing the release of nucleocapsid into the cytosol. **(3)** Following uncoating and genomic RNA release, the non-structural proteins are translated as polyproteins denominated P123 and P1234. **(4)** A replicative complex (RC) formed by uncleaved P123 plus nsP4, the genomic RNA, and several host factors is targeted and anchored at the plasma membrane inducing bulb-shaped invaginations, known as spherules, where RNA synthesis will occur. dsRNA indicates the viral replicative intermediate. nsP1-3 associates with nsP4 in a specific quaternary structure converts the RC into a positive-strand RNA replicase, which synthesizes the viral genomic and subgenomic RNAs. Spherules are internalizate and shape functional large cytopathic vacuoles that bear multiple spherules. **(5)** Subgenomic RNA (26S) is translated, producing the structural polyprotein **(6)** E1and E2-E3 (pE2) are translocated into the ER and go through the post-translational process of maturation and glycosylation. **(7)** Capsid autoproteolysis releases free capsid into the cytoplasm that interacts with genomic RNA, giving origin to the nucleocapsid. **(8)** The viruses bud out of infected cells through the cell membrane in a pH and temperature-dependent process. **(9)** CHIKV replication induces ER stress and activates the Unfolded Protein Response (UPR). By non-elucidated mechanisms CHIKV infection also results in oxidative stress, generating Reactive Oxygen Species (ROS) and Reactive Nitrogen Species (NRS). **(10)** Both ER and oxidative stress can trigger autophagy, a pro-survival signal, in an attempt to preserve cell viability. When CHIKV capsid is produced in the cytoplasm, it can be ubiquitinated and sequestered by adaptor protein SQMT1/p62 into the autophagosomes, leading to capsid degradation in the autophagolisosome. **(11)** CHIKV is able to trigger NLRP3 inflammasome, starting a signaling cascade that culminates in the activation of the caspase 1, that turns able to cleaves of pro IL-1β and pro IL-18, generating mature cytokines, that will elicit adaptive responses, but also can contribute to pathological inflammatory events such as edema and arthritic disease symptoms.

The full-length P1234 is autocatalytically cleaved into nsP4 and P123, the premature cleavage of nsP4 has a simple biological explanation: the cycle’s continuity depends on fast replication of the viral genetic material. The nsP1-4 are part of the replication complex (RC), which will determine the replication of the viral genomic RNA and the transcription of the genomic and the subgenomic (26S RNA) viral RNAs. The initial RC complex is formed by the uncleaved P123 plus nsP4 (P123-nsP4), which is targeted and anchored to the plasma membrane by the association of the nsP1alpha-helical peptide and palmitoylated amino acids within the P123. The association of the nsP1 membrane-binding domain with the plasma membrane will induce bulb-shaped invaginations, called spherule, where viral RNA synthesis takes place (Figure 2). The negative-strand RNA bears the subgenomic promoter, a sequence of 21 nucleotides, complementary to the nucleotides of the junction region, 19 of the upstream and two downstream of the replication’s initiation point. The subgenomic 26S RNA is identical in sequence to the one-third of the genomic RNA 3′ terminus and serves as a template to structural proteins synthesis. Like genomic RNA, the subgenomic RNA is also capped and polyadenylated (Knipe et al., 2001). As P123 is cleaved into the final nsP1, nsP2, and nsP3 proteins, its association with nsP4 in a specific quaternary structure convert the RC into a positive-strand RNA replicase, which will synthesize the viral genomic and subgenomic RNA.

The nsP1 is an initiation factor for negative-strand RNA synthesis and RNA capping via its guanine-7-methyltransferase and guanylyltransferase enzymatic activities.

The nsP2 works as an RNA helicase, a nsPolyprotein protease, and recognizes the subgenomic RNA promoter.

The nsP3 acts as a replicase unit and also as an accessory protein involved in RNA synthesis by recruiting several host-cell factors that participate and optimize viral replication. The nsP3 hypervariable domain (HVD), at the C-terminus, binds the Ras-GHP SH3 domain (G3BP) protein family to promote replication for several alphaviruses. This biding is particularly critical for CHIKV and is, in part, related to the capacity of the virus to inhibit stress granule formation (Kim et al., 2016; Meshram et al., 2018). In this sense, nsP3 VHD binding to the fragile X syndrome (FXR) family members also plays a role in alphavirus replication. Beyond a role in avoiding the formation of stress granules, binding of nsP3 to these proteins is also important to promote viral RNA synthesis by facilitating the assembly of the RC complexes. Different studies have shown that for several alphaviruses the nsP3 binding to these family members is virus-specific and also cell type-specific, presenting a high level of redundancy. However, for CHIKV the binding of host factors from different families is not redundant (Kim et al., 2016; Meshram et al., 2018), pointing out to a critical role of this replication step for the CHIKV–host coevolution.

More recently, two other cellular factors binding to the HVD of nsP3 were implicated in promoting virus replication and permissiveness of CHIKV infection. The host DHX9 DEXH-box helicase is a DNA/RNA helicase that has been demonstrated to participate in the replication of diverse RNA positive viruses (*Picornaviridae*, *Arteriviridae*, *Flaviviridae* – Pestivirus genus, and *Retroviridae* – HIV-1). Matkovic et al. (2019) showed that the nsP3 HVD binds DHX9, redirects this protein from the nucleus to the cytoplasm at discrete puncta structures to increase CHIKV genomic RNA translation early at the viral infectious cycle. Further, they demonstrated that CHIKV nsP2 also binds to DHX9 and targets it to the proteasomal for its degradation. This step is critical to the switch of genomic RNA translation to replication (Matkovic et al., 2019).

Four and a half highly conserved LIM1 domain (FHL1) is a cellular protein that recently has been implicated as a cellular factor promoting CHIKV tropism. This protein has three distinct spliced isoforms in human cells (1A, 1B, and 1C). 1A is abundantly expressed in skeletal muscles and fibroblasts, while 1B and 1C are present in muscle, brain, and testis. Meertens et al. (2019) demonstrated that FHL1 binds to the nsP3 HVD and promotes CHIKV replication. This host factor was also important to the ONNV Old World alphavirus, while it had no impact on the replication of the New World alphaviruses MAYV, SINV, and Semliki Forest Virus (SFV). Primary cells from patients with FHL1 deficiency were resistant to CHIKV infection, highlighting the importance of this cellular factor in promoting skeletal muscle and fibroblast tropism of CHIKV and viral pathogenesis. Strikingly, the dependence of this factor was demonstrated for all CHIKV strains, except the Western African linage, reinforcing the hypothesis that the success of emergent and re-emergent CHIKV strains to spread and establish in the human population and on mosquito vectors will be determined by the interaction of different host factors and the viral proteins.

Collectively, these new findings help to expand the model of CHIKV replication: after the release of viral capsid in the target cell cytoplasm, uncoating of genomic RNA is followed by the translation of P123 and P1234 non-structural precursors, facilitated by the host DHX9 helicase. The initial RC complex formed by P123 and nsP4 then associates with the incoming genomic RNA and the complex is targeted to the plasma membrane by the nsP1 portion of P123. G3BP and FXR factors associates with the RC complex at this very early stage to avoid genomic RNA degradation. DHX9 degradation by the viral nsP2 is critical to the switch from translation to viral replication. Once the first double-stranded RNA replication intermediates are synthesized, they are isolated into the membrane spherule leading to the amplification of these processes. The new synthesized positive-stranded genomic RNAs exit the membrane spherules and are translated in close proximity of the plasma membrane, forming new RC complexes, which by binding of G3BP, FXR, and possibly FHL1, oligomerize and increase the formation of new RCs to amplify the amount of viral genomic RNA within the infected cell early on the infection. Studies from SINV and SFV suggest a high dynamics of spherule internalization through a Phosphatidylinositol-3 kinase (PI3K) activated endocytosis, actin and myosin-dependent transport, and fusion with late endosomes (Spuul et al., 2010), leading to the formation of the so-called large cytopathic vacuoles (CPV-1) (Figure 2).

Subgenomic viral RNAs exiting from CPV-1 are immediately translated in close proximity to the Endoplasmic Reticulum (ER) to produce the viral structural polyprotein. At the C-terminal of the CP, a peptide signal leads to the translocation of the polyprotein across the ER membrane. Whereas, through proteolytic processing, it will give rise to intermediate proteins CP, p62, 6K or 6k/TF and E1. From a new stage of proteolysis, hijacking cellular proteases, the final structural proteins will appear: CP, E2, E3, 6K, or 6K/TF and E1 (Aliperti and Schlesinger, 1978; Kääriäinen and Ahola, 2002; Melton et al., 2002; Ramsey and Mukhopadhyay, 2017). Alphavirus capsid proteins are multifunctional and have an intrinsic protease activity. Thus, CP is autocleaved out of the structural precursor protein by its Serine-protease activity. In CHIKV the CP N-terminal is unstructured and has the RNA-binding domain, whereas the C-terminal globular domain harbor the Serine-Histidine-Aspartic acid protease domain. CP will remain in the cytosol for the formation of the viral nucleocapsid.

The glycoprotein E1 has only one transmembrane domain, while E2 has two transmembrane domains. They go through a post-translational process of maturation and glycosylation and are exported in vesicles, hijacking the cellular secretory machinery, up to the cell’s plasma membrane.

The glycoprotein E3 is translated right after the capsid protein; it aids with cellular chaperones in the proper folding of E2 and E1, and has a specified signal sequence that addresses the remainder of the polyprotein to the ER membranes. It remains associated with E2, which is why both are called pE2 at this stage, until the moment it reaches the *trans*-Golgi, where the cellular Furin protease is responsible for the cleavage of pE2 in E2 and E3, making the “spike” now functional.

The 6K protein is a hydrophobic small protein that joins the E2 and E1 parts of the polyprotein, allowing for proper envelope processing. It also participates in membrane permeabilization, virus assembly, and budding. An additional protein, which is an extension of the 6K N-terminus, is also synthesized during alphavirus infection. This protein results from a −1 frameshift event 40 nucleotides before the beginning of E1 glycoprotein and leads to the formation of a truncated structural precursor, as described above (Firth et al., 2008; Snyder et al., 2013). This frameshift occurs in a 10–18% frequency during the subgenomic RNA translation. The resulting protein is an 8 kDa TF that is incorporated into viral particles and probably participates in viral assembly.

The newly formed virus particles bud out from infected cells through the cell membrane in a pH and temperature-dependent process, which requires that the temperature is close to physiological (∼36°C) and that the pH is neutral or slightly alkaline (Lu and Kielian, 2000). There are some other mandatory requirements for exporting viral particles, such as the connection between the capsid and E2 (Suomalainen et al., 1992), the heterodimerization between E1 and E2 (Sjöberg and Garoff, 2003), and the interaction between virus’ structures and host-cell factors: Arf1 and Rac1 assisting the stabilization of E2/E1-containing cytopathic vacuole type II, trafficked by actin filaments—that E2 apparently induces the accumulation and the elongation—by a mechanism involving Rac1, Arp3, and PIP5K1, all constitutive cellular factors (Radoshitzky et al., 2016). Figure 2 summarizes the major features of the CHIKV replication cycle.

## Virus–Cell Interaction

### CHIKV Infection and Host and Virus Transcriptional and Translational Regulation

Transcriptional shutoff during CHIKV infection impairs the cellular response to viral replication and avoid the establishment of an antiviral state. The CHIKV nsP2 mediate degradation of RBP1, the catalytic subunit of cellular RNA polymerase II, resulting in transcriptional shutoff, cytopathic effect, and reduced IFN-β production. Thus, nsP2 expression is cytotoxic and suppresses both cytokine production and activation of interferon-stimulated genes (ISGs) in infected cells (Akhrymuk et al., 2019).

CHIKV infection also results in the shutoff of host cell protein synthesis, whereas viral proteins continue to be synthesized. The host cell shutoff is a result of Eukaryotic Translation Initiation Factor 2 α (eIF2α) phosphorylation (White et al., 2011). Phosphorylation of eIF2α disables the ternary complex, essential for cap-dependent translation initiation. How CHIKV infection results in eIF2α phosphorylation remain unclear. Although infection increases the double-stranded RNA-dependent protein kinase (PKR) activation, eIF2α phosphorylation also occurs independently of PKR (White et al., 2011).

Moreover, CHIKV modulates protein synthesis by interfering with mTOR activation. Joubert et al. (2015) demonstrated that during the first 24 h of infection, mTOR and S6K phosphorylation is reduced, which directly impacts on host cell protein synthesis. mTORC1 low activity is associated with AMP phosphorylation kinase (p-AMPK), an energy-sensing enzyme, followed by TSC2 activation, which acts as an inhibitor of mTOR phosphorylation (Joubert et al., 2012). Inhibition of the mTOR complex 1 (mTORC1) increases CHIKV production and this effect is independent of IFN-I production and autophagy induction. To bypass the deleterious effect of mTORC1 inhibition for cap-dependent mRNA translation, CHIKV protein synthesis is mediated via Mnk/eIF4E pathway (Joubert et al., 2015). Interestingly, mTORC1 inhibition also increases SINV infection, but had no effect on influenza A infection (a member of the *Orthomyxoviridae* family), suggesting that different viruses developed singular strategies to modulate mTORC1 activity (Joubert et al., 2015).

The PI3K-AKT-mTOR pathway is the major pathway that mTOR is involved in. Thaa et al. (2015) demonstrated that CHIKV infection induces AKT serine 473 phosphorylation but had no effect on S6 phosphorylation, one of the downstream targets of the PI3K-AKT-mTOR pathway. AKT phosphorylation by CHIKV is lower compared with other alphaviruses like SFV. SFV nsP3 triggers strong AKT activation, which is associated with the RC internalization. On the other hand, replication complexes were broadly localized at the cell periphery in CHIKV infection (Thaa et al., 2015). However, it remains to be elucidated how different CHIKV strains will impact on both AKT activation and mTOR modulation. Different alphaviruses modulate the PI3K-AKT-mTOR pathway in specific manners associated with particular virus replication features.

### CHIKV, Autophagy, and Oxidative Stress

Macroautophagy, referred herein as autophagy, is a homeostatic process conserved in eukaryotes that recycle cargo proteins and organelles through lysosomal degradation by their selective sequestration inside double-membrane vesicles, known as autophagosome (Yang and Klionsky, 2010). It is also described as a cytoprotective process with important roles in immunity response against sterile and infection-associated inflammation, including viral infection (Deretic and Levine, 2018).

Despite its relevance to the immune response against infections, autophagy may play a role in both anti and pro-viral replication. For instance, some viruses are able to subjugate the autophagy machinery in its own advantage. This process has been investigated for alphaviruses (Liang et al., 1998; Orvedahl et al., 2007, 2010; Eng et al., 2012; Joubert et al., 2012). The role of autophagy during CHIKV infection is still controversial and can be divergent according to the cell type used to replicate CHIKV.

First reports showed that CHIKV infection of human embryonic kidney 293 cells (HEK-293T) leads to an increased number of the microtubule-associated protein 1A/1B light-chain 3 (LC3) puncta and augmentation of membrane-bound vacuoles, suggesting that CHIKV infection triggers an autophagic response (Krejbich-Trotot et al., 2011). Accordingly, CHIKV replication was dramatically reduced when autophagy was blocked biochemically or by RNA interference (Krejbich-Trotot et al., 2011).

Oxidative stress is an important mechanism to fight back pathogens. It occurs due to a dysregulation of redox control, caused by increased levels of reactive oxygen species (ROS) and reactive nitrogen species (RNS) and/or a reduction in the antioxidant defense system (Jones, 2006; Cataldi, 2010). Free oxidative species are able to initiate autophagy and can also lead to cell death during strong and prolonged stimulation (Djavaheri-Mergny et al., 2007; Filomeni et al., 2010). Joubert et al. (2015) assessed CHIKV capacity to induce ROS and RNS. They observed, in murine fibroblast cells (MEF), that CHIKV infection led to increased production of both ROS and NO. In addition, they demonstrated that CHIKV-induced autophagy on these cells was mediated by the independent induction of endoplasmatic reticulum (ER) and oxidative stress pathways, delaying cell death by apoptosis through induction of IRE1a-XBP-1 pathway at the same time as ROS-mediated AMPK activation and mTOR inhibition. Consequently, the treatment with *N*-acetyl-l-cysteine, a potent antioxidant, reduces CHIKV-induced autophagy, observed by the decrease in LC3 puncta on these cells (Joubert et al., 2012). Therefore, it was demonstrated that CHIKV infection can induce endoplasmic reticulum and oxidative stress at the early stages of infection to trigger autophagy (Figure 2).

Interestingly, during the late stages of viral replication in MEF cells, autophagy is suppressed concomitantly with enhanced cell death by apoptosis, favoring viral release and spread (Joubert et al., 2012), showing a time-dependent pattern of autophagy regulation by CHIKV infection.

In human epithelial adenocarcinoma cells (HeLa), CHIKV infection can regulate autophagy through the interaction between viral proteins and the autophagic receptors sequestosome 1/p62 (SQSTM1/p62) and calcium-binding and coiled-coil domain-containing protein 2/nuclear dot 10 protein 52, known as NDP52. Both proteins are able to interact with both cargo proteins and LC3, directing autophagy targets to autophagosomes (Judith et al., 2013). It was shown that SQSTM1/p62 can protect CHIKV infected human cells from death by binding ubiquitinated viral capsid and targeting it to lysosomal degradation (Figure 2). Moreover, CHIKV infection in certain cell types leads to robust SQSTM1/p62 degradation. Differently, it is being described that NDP52, but not its murine ortholog, interacts with the viral protein nsP2 promoting viral replication (Judith et al., 2013). Therefore, during CHIKV infection, autophagy can be regulated in different ways playing both pro- or anti-viral roles according to the time of the replication cycle and to the cell type and this can be crucial for the infection progression and virus spread.

### CHIKV and the Endoplasmic Reticulum Stress

The ER is an essential cellular membrane organelle, with a dynamic structure that plays important roles in many cellular processes, including protein synthesis, folding and secretion, calcium homeostasis, lipid production, and the transport of cellular components. ER plays an essential role in the replication process of several viruses, including viral entry, assembly, protein synthesis, and genome replication. The massive viral replication can cause disturbances on the protein folding machinery, disrupting ER homeostasis, which culminates in ER stress (Liu and Kaufman, 2003; He, 2006; Inoue and Tsai, 2013; Jheng et al., 2014). The ER stress activates an evolutionarily conserved prosurvival pathway, termed the unfolded protein response (UPR), that acts for maintenance of ER homeostasis. UPR has three main mechanisms to restore the adequate ER function: (1) inhibition of protein synthesis, (2) induction of genes of chaperone family, necessary for the folding protein processes, (3) eliminating the amount of misfolded or unfolded proteins by activation of the ER-associated protein degradation (ERAD) pathway (Malhotra and Kaufman, 2007; Hetz et al., 2011).

In mammalian cells, the three main branches of the UPR are the protein kinase-like ER-resident kinase (PERK), the activating transcription factor 6 (ATF6), and the inositol-requiring enzyme 1 (IRE1). These proteins are associated with the ER chaperone BiP/Grp78. When unproperly folded proteins accumulate in the ER lumen, BiP/Grp78 dissociates from these three transmembrane signaling proteins, resulting in activation and initiation of the UPR pathway. Then, activated PERK phosphorylates eIF2α at Ser51, decreasing the load of proteins entering into the ER lumen by blocking general protein translation. Activated ATF6 is a transcription factor that increases the transcription of a number of ER chaperones, the X box-binding protein 1 (XBP1), and other transcription factors. Activation of IRE1 results in the IRE1 mediated splicing of the XBP1 mRNA, which activates the expression of downstream genes like chaperones and other proteins involved in protein degradation (Yoshida et al., 2001; Harding et al., 2002; Vattem and Wek, 2004; Jheng et al., 2014).

Beyond triggering ER stress and UPR, viruses have evolved different strategies to subvert these cellular responses for their own benefit, e.g., enhancing replication, persisting in infected cells, and evading immune responses, as described for several viral families, such as *Flav* i-, *Herpes*-, and *Togaviridae* (reviewed by Ambrose and Mackenzie, 2011; Green et al., 2014; Li et al., 2015).

CHIKV infection results in the activation of the UPR pathway in different cell lines. However, results from different groups are discordant and may reflect the cell-specificity for UPR activation. Fros et al. (2015) showed that in Vero cells, the expression of CHIKV envelope proteins alone can induce UPR by the upregulation of ATF4 and GRP78/Bip. Additionally, CHIKV-infected Vero and an adult WT mouse model of CHIKV arthritis only partially induced by XBP1. Furthermore, the authors demonstrated that individual expression of CHIKV non-structural protein nsP2 protein was sufficient to inhibit the UPR pathway (Fros et al., 2015). Whereas, CHIKV infection of HEK293 cells activated the ATF6-UPR branch, but not IRE1 or PERK pathways. In these cells, CHIKV infection blocked eIF2α phosphorylation even in the presence of pharmacological activation of UPR by Thapsigargin and Tunicamycin. The authors demonstrated that nsP4 was sufficient to inhibit phosphorylation of eIF2α (Rathore et al., 2013).

ER stress, autophagy, and apoptosis in response to CHIKV infection were also investigated in HeLa and HepG2 cells and showed distinct results. In HeLa cells, CHIKV infection activated the PERK branch of UPR, with consequent eIF2α phosphorylation (Khongwichit et al., 2016). Diversely, Joubert et al. (2012) observed activation of UPR in HeLa through the splicing of XBP1 by IRE1 during CHIKV infection. The ATF6 branch was also activated in these cells. Whereas in HepG2 IRE1 activation was strong, the activation of PERK and ATF6 was less pronounced and only a low level of eIF2α phosphorylation was observed. For both cells, the downstream protein CHOP, which is involved in apoptosis signaling, was also upregulated (Khongwichit et al., 2016).

Moreover, the silencing of IRE1 during CHIKV infection of HeLa leads to fewer CHIKV-induced autophagosomes. Apparently, CHIKV-induced autophagy is dependent on both triggering of oxidative stress and UPR pathways. These data reinforce the idea that the ER could serve as a subcellular platform for autophagy initiation. Signaling of UPR and autophagy are interconnected, and these two pathways crosstalk to modulate the cell survival or dead by apoptosis (Bernales et al., 2006; Axe et al., 2008; Joubert et al., 2012).

Data regarding ER stress and UPR during CHIKV infection, although apparently conflicting, indicate that CHIKV infection can elicit distinct interactions with cell machinery depending on the cell type and possibly the viral strain analyzed. These data raise the necessity to further investigate the role of UPR on cell lines with close similarity to the cells naturally infected by CHIKV, as epithelial cells, skin fibroblasts, muscular, and endothelial cells. Furthermore, the use of mouse models of infection can also contribute to determining the relevance of the UPR signaling to CHIKV replication and pathogenesis.

### CHIKV and the Inflammasome

Inflammasomes are cytosolic molecular complexes that initiate inflammatory responses upon the detection of pathogens, cellular damage, or environmental irritants by the pattern recognition receptors (PRRs). Upon activation, inflammasome is assembled and activates caspase-1, which cleaves proinflammatory cytokines prointerleukin-1β (proIL-1β) and prointerleukin-18 (pro IL-18) resulting in proteolytic maturation and secretion of active forms of these cytokines (IL1- β and IL-18, respectively). All these signaling cascades lead to a type of programmed cell death known as pyroptosis that is inherently inflammatory and characterized by caspase 1-dependent formation of plasma membrane pores leading to ion fluxes, that culminates with the cytoplasmic membrane rupture and subsequent release of intracellular content in order to control microbial infections (Martinon et al., 2002; Bergsbaken et al., 2009; Conforti-Andreoni et al., 2011; Figure 2).

In a scenario of viral infections, inflammasome can amplify the sensing of viral nucleic acids (RNA or DNA). Although inflammasome signaling and activity is supposed to resolve the infection and promote homeostasis, high levels of inflammasome-triggered proinflammatory cytokines have been associated with inflammation and pathogenesis of several viral, bacterial, autoimmune diseases, and cancer (Davis et al., 2011; McAuley et al., 2013; Negash et al., 2013; Wikan et al., 2014; Olcum et al., 2020).

The role of inflammasome on CHIKV replication and pathogenesis has been poorly explored. One study, from Ekchariyawat et al. (2015), demonstrated that CHIKV infection could generate inflammasome signaling in human dermal fibroblasts cells, culminating in activation of caspase 1 and increase IL1 β expression and maturation, as well as induction of the expression of the inflammasome sensor AIM2, although AIM2 has been implicated in recognition of dsDNA only. In the absence of inflammasome assembly (through caspase 1 silencing), CHIKV replication rates were enhanced (Ekchariyawat et al., 2015). Moreover, ASC2 and NLRP3 expression, as well as IFN- β and some ISGs, were upregulated in CHIKV-infected fibroblasts.

More recently, Chen and colleagues showed that NLRP3 inflammasome is activated in humans and mice. Expression of NLRP3, ASC, and caspase 1 was100-fold enhanced in PBMCs from a cohort of CHIKV-infected patients. Also, IL18 and IL1 β mRNA levels were increased in these patients in the acute phase of CHIKF (Chen et al., 2017). In a mouse model of CHIKV-induced inflammation, subcutaneous inoculation of ECSA CHIKV strain isolated from La Réunion (LR2006-OPY1), a microarray gene analysis revealed increased expression of NLRP3, NLRP1, NLRC4, IL-1β - and IL-18-binding protein, caspase-1, IL-18 receptor, and IL-18 receptor accessory protein, with high expression coinciding with the peak of inflammatory arthritic disease symptoms (Chen et al., 2017). Furthermore, using a molecule that inhibits the activation of the NLRP3 inflammasome, the group observed substantial improvement of arthritic symptoms, with a reduction of inflammation, myositis, and osteoclastic bone loss, although the general replication remained at the same levels. Also, in ASC^–/–^ mice the foot swelling after CHIKV infection was less severe, compared to wild type mice. Taken together, these studies reveal the relevance of inflammasome on CHIKV infection, highlighting its role in the pathology of arthritic disease and inflammation. Concisely, the compelling data open the possibility for the development of therapeutic strategies targeting the inflammasome pathway to ameliorate arthritic symptoms.

### CHIKV Pathogenesis

Dermal fibroblasts are the primary targets and the main sites of CHIKV replication (Sourisseau et al., 2007; Ekchariyawat et al., 2015), but other skin cells are also susceptible, like keratinocytes and melanocytes (Gasque and Jaffar-Bandjee, 2015). From the skin, the virus migrates via lymphatic circulation, to the nearest lymph node, reaching the bloodstream where it infects mostly monocyte-derived macrophages (Sourisseau et al., 2007). In a non-human primate (NHP) model, CHIKV migration was demonstrated by the presence of CD68^+^ macrophages positive for CHIKV antigen trafficking to lymphoid tissue and the spleen from early timepoint up to 3 months after infection (Labadie et al., 2010). From the blood, the virus reaches joints, muscles, and bones, which are the sites most linked to the chronic symptoms of the disease. Satellite cells of skeletal muscle are permissible for CHIKV infection and can act as a reservoir of mature skeletal fibers precursors, therefore, they have an active and crucial role in maintaining tissue structure (Ozden et al., 2007) and, when infected, can constitute a site of viral persistence. Mature skeletal muscle fibers and primary myoblasts have also been targeted by CHIKV (Couderc et al., 2008; Lohachanakul et al., 2015). In the joints, viral RNA and proteins were found during the acute and chronic phase of the infection; macrophages, primary human chondroblasts, and fibroblasts from synovial tissues are susceptible to CHIKV infection, with synovial macrophages being the main site of viral persistence linked to CHIKV (Hoarau et al., 2010; Zhang et al., 2018). The bones of the regions closest to the joints are also targets of infection since primary human osteoblasts are permissive to CHIKV (Chen et al., 2015). These are the preferred targets of viruses, which are not coincidentally linked to the most commonly observed clinical manifestations. The appearance of unusual clinical manifestations, affecting central nervous, cardiovascular, respiratory, digestive, hematopoietic, and renal systems is due to the presence of cells, vital to local homeostasis, that is also susceptible to the CHIKV infection.

### The Immune Response at Acute Phase of Infection

The type I interferon (IFN) response is an early innate immune mechanism that elicits antiviral responses and activates components of the innate and adaptive immune systems. IFNs are quickly induced after recognition of viruses by host pattern recognition receptors (PRRs), mainly by Toll-like receptors (TLRs), cytosolic receptors as retinoic acid-inducible gene-I (RIG-I), and melanoma differentiation-associated gene 5 (MDA5) (Thon-Hon et al., 2012; Jang et al., 2015). After recognition of their respective ligands (double-stranded [ds] RNA for RIG-I and MDA5), the mitochondrial antiviral-signaling protein (MAVS) is activated via Card-card interactions, domains presented both in MAVS and cytosolic receptors. Then, TBK1 is activated by MAVs and phosphorylates the interferon regulatory factor 3 (IRF-3), which dimerizes and translocates into the nucleus. This signaling pathway induces the production of type I IFNs through activation of the IFN-α/β promoter. IFNs are secreted and act in autocrine and paracrine ways, after activation of the interferon-α/β receptor (IFNAR), triggering a signaling cascade of events that culminates in the expression of ISGs that enhance viral recognition and interfere with several steps of the viral cycle (Platanias, 2005; Hu et al., 2018).

The role of IFNs for CHIKV pathogenesis is well known. Viral replication is controlled by IFNs in cells, and mice lacking IFNAR have important viral dissemination, related to high rates of mortality (Schilte et al., 2010; Suhrbier et al., 2012). In cynomolgus macaques, infection with the isolate CHIKV-LR recapitulates common characteristics of the immune response, such as an increase in plasma levels of IFN-α/β, interleukin 6, and monocyte chemoattractant protein 1, correlating with peak levels of viremia (Labadie et al., 2010). Additionally, in fibroblastic cell lines, CHIKV infection induces the expression of antiviral genes, as IFN-α and RIG-I. Moreover, CHIKV is able to interfere with the nuclear translocation of phosphorylated STAT1, a transcription factor that promotes the expression of several ISGs (Thon-Hon et al., 2012).

Cook et al. (2019) recently showed distinct but synergistic roles for IFN-α and β in controlling CHIKV replication and disease. While IFN-α acts in non-hematopoietic cell types, reducing replication and early dissemination of CHIKV, IFN-β has a substantial impact on pathogenesis, since it can limit neutrophil-mediated inflammation at the site of infection (Cook et al., 2019).

Recently, Bae et al. (2019), through a gene screening in HEK293T cells, reported that viral protein nsP2 and envelope glycoproteins E1 and E2 are strong antagonists of the IFN-β signaling pathway. Triggering of IFN response, although a common feature of RNA viruses, can vary in amplitude and intensity depending on the virus species and even different genotypes and/or strains from the same species. The characterization of IFN response during the infection of the CHIKV isolates related to the most recent epidemics in Latin America will allow us to understand the pathogenic potential of these viruses.

Natural killer (NK) cells are at the front line in controlling virus replication via stimulation of IFN-I. Like other viruses, CHIKV is able to induce the activation of a phenotype rarely seen in the NK cells of healthy patients; these cells have the NKG2C1 receptor activated, which makes them highly cytotoxic, leading to the lysis of infected cells (Petitdemange et al., 2011).

Antibodies and CD8^+^ T cells are key players in adaptive immune responses. It has been shown the activation and multiplication of CD8^+^ T cells during the first days of infection followed by a switch to CD4^+^ T-cells, but the exact role of T-cells in CHIKV infection remains uncertain. In mice, CD8^+^ T cells were recruited to the musculoskeletal tissue in the first week of infection (Teo et al., 2013), which could be one of the reasons for the increased levels of IFN-γ (Wauquier et al., 2011). These cells can also be linked, among other mechanisms described above, with the control of viral replication in the acute phase, since there is an increase in perforins, granzymes, and proteins linked to the degranulation of CD8^+^ T cells, which would culminate in apoptosis of infected cells (Dias et al., 2018).

Regarding antibodies, anti-CHIKV antibodies are fully capable of offering protection even in the first days of infection, since IgM is detected initially at 2–3 days after the appearance of symptoms (Litzba et al., 2008). Antibody-mediated response suppresses the spread of the virus, either by direct neutralization or by activation of the complement system (Lum et al., 2013). In a study with rhesus macaques comparing the CHIKV strains La Reunion (CHIKV-LR) and Western Africa 37997 (CHIKV-37997), T-cell and antibody responses were more robust in the animals infected with LR compared to 37997 (Messaoudi et al., 2013). A different study showed that 90% of antibody response against CHIKV was mediated by IgM within the first 9 days of infection in cynomolgus macaques inoculated with CHIKV-LR (Kam et al., 2014).

### Immune Response at the Chronic Phase of Infection

Chronification of the infection usually leads to continuous inflammation of the joints. This inflammation can be immune-mediated by several elements that, *a priori*, could be allies in fighting infection; it is possible for NK cells to infiltrate synovial tissues and maintain an inflammatory environment conducive to arthralgia, for example. However, NK cells associated with the chronic phase of the disease have reduced expression of cytolytic mechanisms, such as perforin, and increased expression of IFN-γ and TNF-⟨, pro-inflammatory components that can contribute to the establishment of a highly inflamed environment in joints (Thanapati et al., 2017).

### The CHIKV-Induced Disease

#### Usual Clinical Manifestation of CHIKF

##### Arthritis and arthralgia

CHIKV, among other mosquito-transmitted alphaviruses, like RRV, Barmah Forest Virus (BFV), and MAYV, can cause debilitating pain and inflammation of joints in humans (Staples et al., 2009), leading to the severe and debilitating rheumatic symptoms that are experienced by most infected individuals, that could result in a negative impact on everyday activities (Ross, 1956). For this reason, epidemiological studies established unusually severe joint pain as the distinguishing and most common feature of CHIKV infection (Brighton et al., 1983; Powers and Logue, 2007). The severe pain starts in the acute phase of infection, affecting both peripheral and large joints, and becomes chronic, typically lasting from weeks to months (Queyriaux et al., 2008; Vijayakumar et al., 2011). In 25–42% of infections, inflammatory-related affections, like joint effusions, redness, and warmth, can be observed. These joint symptoms are usually polyarticular, bilateral, symmetrical, and can fluctuate, but the anatomical location does not usually change (Deller and Russell, 1968; Queyriaux et al., 2008; Simon et al., 2011; Vijayakumar et al., 2011).

##### Fever

One of the most common symptoms of the acute phase of infection is an abrupt onset of fever, coincident with the viremia and polyarthralgia, reaching 40°C in some cases, resulting in chills and rigors (Simon et al., 2011). Fever, in addition to lasting from many days to 2 weeks, are also typically biphasic in nature (with a period of remission of 1–6 days) (Halstead et al., 1969; Thiberville et al., 2013), which means an early elevation in body temperature followed by a later one, caused by a dynamic balance between exogenous and endogenous pyrogens and prostaglandins.

##### Myalgia

Muscle pain, dissociated from inflammation (myositis), is frequent in 46–59% of cases, mainly affecting arms, thighs, and calves (Zim et al., 2013). It can be a confounding factor, since other arbovirus diseases, such as dengue, can also develop myalgia (Kumar et al., 2017), one of the reasons why some researchers call CHIKV clinical manifestations as a “dengue-like” disease, but with a particular articular tropism.

##### Dermatologic involvement

The most common cutaneous manifestation of CHIKF is macular or maculopapular rash, distributed mainly in the extremities, trunk, and face, associated with severe pruritus (Shivakumar et al., 2007), observed in up to 50% of cases. In most cases, the lesions follow fever episodes, but they also can occur concomitantly since both depend on viremia. They generally do not produce sequelae, but, in some patients, they induce pigmentary changes, mainly in the malar area of the face, with a predilection for the tip of the nose, but also seen in extremities and trunk, desquamation and xerosis (Prashant et al., 2009). There are other less common dermatological manifestations, which includes erythema and swelling of the pinnae, mimicking erysipelas’ Milian ear sign; multiple aphthae, erosions, and cheilitis were also observed in oral mucosa, but they were all no-sequelae self-limited manifestations, except for hyperpigmentation of the hard palate in a few patients; and genital involvement, in the form of ulcers, over the scrotum and base of the penile shaft in men and labia majora in women. The infection can also flare-up of pre-existing psoriasis and lichen planus manifestations.

##### Other usual manifestations

Pain in the ligaments, headache, fatigue, and severe tiredness, digestive symptoms (diarrhea, vomiting, gastrointestinal bleeding, nausea or abdominal pain), red eyes, conjunctivitis, and lymphadenopathy have also been described, only during the acute phase of infection (Economopoulou et al., 2009); therefore, the impact on the quality of life of people affected by the infection begins with the first symptoms and extends to the remission of polyarthralgia at the end of the chronic phase.

#### Unusual Manifestations of CHIKV Infection

Atypical manifestations of the infection, unlike the aforementioned typical manifestations, depend mostly on the underlying disease, already manifested and exacerbated by the infection or only predisposing in the affected individual, and in this case, CHIKV can be a trigger for the onset of its clinical syndrome. Of note, the spread of new epidemic strains has the potential to induce new subsets of clinical manifestations.

##### Neurological complications

In both adults and children, the most prevalent neurological manifestation is encephalitis, during the acute phase of the infection, usually manifested in less than 24 h after the sudden onset of high fever (Robin et al., 2008; Venkatesan et al., 2013). Although the manifestation of encephalitis, in general, is not related to the age of the patient, the incidence of CHIKV-associated encephalitis shows that individuals younger than 3 years old or older than 65 are more likely to develop the syndrome. Retrospective studies have made it possible to estimate a frequency of 8.6 per 100,000 CHIKV infection cases (Simon et al., 2007). Epileptic seizures, meningoencephalitis, syndrome of meningeal irritation and Guillain-Barré syndrome have also been described, but these are considerably less frequent cases (Robin et al., 2008; Tournebize et al., 2009; Venkatesan et al., 2013; Gérardin et al., 2016); further studies still need to address whether the unprecedented epidemics of CHIKV infection in the South American continent was in fact accompanied by a higher frequency of higher morbidity and atypical clinical manifestations. Some reports, however, had already associated CHIKV infection with diverse neurological complications (Pereira et al., 2017; Mehta et al., 2018).

##### Cardiovascular manifestations

Heart failure was diagnosed in patients with acute infection during La Réunion (island) outbreak of chikungunya fever, in 2005–2006 (Robin et al., 2008), but approximately 60% of the cases have a previous cardiovascular pathological history, such as valvular or coronary disease. This scenario allows us to jump to two conclusions: (1) 40% of infected patients had a flaw in one of their most vital systems without first manifesting any symptoms that involved it, which makes CHIKV infection a potential cardiovascular risk factor for healthy patients; and (2) the virus has a potentiating character, that is, it can be an unexpected factor in the prognosis of cardiovascular diseases previously diagnosed. Myocarditis after arboviruses infections has been described since 1972 (Menon et al., 2010), which can be the main cause for other registered manifestations, which include ventricular and atrial gallops, tachycardia and tachypnea, blood pressure instability, chest pain, electrocardiograph (ECG) abnormalities, and acute myocardial infarction (Spodick, 1986; Dec et al., 1992; Touret et al., 2006).

##### Pregnancy risks and vertical transmission

Although there are reports of concomitance between infection and spontaneous abortions in the second trimester (Dreier et al., 2014), studies have failed to establish a direct relationship between prenatal obstetric complications and CHIKV infection. Regarding its symptoms, on the other hand, the management of infected pregnant women needs to be delicate, since classic high fever can lead to neural tube defects, congenital heart defects, and oral clefts when it occurs in the first trimester of pregnancy (Fritel et al., 2010), and when it occurs in the second and third trimesters, it can result in abrupt uterine contractions and abnormalities in the fetal heart rhythm, resulting in premature births or stillborn babies (Torres et al., 2016). When it comes to mother-to-child transmission, there is no evidence to sustain the antepartum or peripartum risk of fetal transplacental infection and infected newborns are linked only to the intrapartum transmission when the parturient has a positive viremia (Solanki et al., 2007; Gérardin et al., 2008; Sissoko et al., 2008).

##### Renal disorders

An acute pre-renal failure was reported in several cases, of which one-third of the affected patients with previous kidney disease (Robin et al., 2008). The condition is usually controlled by increasing the patient’s blood volume by intravenous hydration, and the reported cases seem to have responded well to this therapeutic approach. There’s only one case of a nephritic syndrome that emerged during an outbreak of CHIKV in Delhi with full recovery (Lemant et al., 2008).

##### Deaths

CHIKV was recognized as a non-lethal infection, however during the outbreak in Reunion Island in 2005–2006, the greater number of patients with atypical manifestations of the infection also contributed to the increase in CHIKV-related deaths, with a mortality rate as high as 48% (Renault et al., 2007). Another study points to a significantly lower rate, of approximately 10% (Economopoulou et al., 2009), but it also links all deaths to the aforementioned atypical manifestations. The major concern of analysts is that many deaths during epidemic periods were underreported by health professionals, which would make the infection mortality rate higher than that already estimated for the disease.

As described above, several atypical manifestations of CHIKV were reported upon recent reemergence and emergence of CHIKV worldwide. In La Reunion Island, clinical features that had never been associated with CHIKF were reported, such as pneumonia, diabetes, bullous dermatosis, toxic hepatitis, encephalitis or meningoencephalitis, myocarditis, and cardio-respiratory failure (Economopoulou et al., 2009). During the 2008 outbreak of CHIKF in South India, various cases of cutaneous manifestations, including vesiculobullous eruptions with significant morbidity in infants were associated with CHIKV infections (Inamadar et al., 2008). The authors hypothesized that these novel manifestations could be associated with the IOL circulating strain of CHIKV. In French Guiana, the introduction of the CHIKV Asian strain was associated with severe forms of the disease, including cases of sepsis and a Guillian-Barrè syndrome (Bonifay et al., 2018). In Brazil, where the ECSA strain predominates, atypical neurological manifestations have been reported (Azevedo et al., 2018). Although it is still early to associate CHIKV infection severity with the introduction of different viral strains in susceptible populations, studies are needed in other to characterize the biological properties of different CHIKV strains.

## Discussion

The introduction of CHIKV within the human population is estimated to have occurred at the beginning of the 20th century; still, the highly epidemic potential of this arbovirus was only truly appreciated after the large epidemics occurring from the first decade of the 21st century in Kenya, La Reunion islands, and the Caribbean. Strikingly, the CHIKV genotype responsible for these large epidemics was the ECSA-derived Indian Ocean Lineage. Mutation within the viral envelope glycoproteins that accounted for virus adaptability to *Ae. albopictus* are regarded as an important factor leading to massive virus dissemination in these regions. However, coincident with this unprecedented spread of CHIKV, descriptions of atypical clinical outcomes began to be reported. In Brazil, a CHIKV ECSA genotype, derived from an ancestral ECSA virus from Central Africa, was responsible for the large epidemic that occurred from 2015 to 2018 in several parts of the national territory affecting at least 700,000 individuals. The severity of the symptoms and the morbidity of CHIKF still need to be accounted, but there are reports of atypical cases of meningoencephalitis and other neurological complications in CHIKV-infected patients in Brazil.

Although the factors involved in the unprecedented dissemination of ECSA-derived IOL could be due to viral determinants related to adaptability to the arthropod vector, as already demonstrated, other viral determinants such as increased viral replication capacity, modulation of host IFN response, that has the potential to increase virus pathogenicity cannot be excluded. In fact, as reviewed here, several studies conducted with the La Reunion CHIKV isolate CHIKV-LR, demonstrated its higher capacity to induce disease symptoms and establish infection in immunocompetent murine models of infection when compared to other CHIKV genotypes. Although in the immunocompromised murine model these results were not reproducible and the ECSA-derived IOL was not able to induce higher mortality rates when compared to the other CHIKV genotypes. This data reinforces the importance of continuous studying CHIKV replication properties, host-cell interaction, and pathogenesis to comprehensively address the epidemic potential of different emerging and reemerging CHIKV genotypes.

The South-American ECSA genotype, on the other hand, does not harbor the vector-adapting mutations observed for the ECSA-derived IOL, and studies are urgently needed to understand the role of unique mutations observed throughout its genome for mosquito adaptability, virus replication, and pathogenesis. Characterization of viral determinants of disease severity and virus pathogenicity in this emerging ECSA-related genotype will help to predict the impact of future epidemics. As well as the characterization of different genotypes of CHIKV in terms of replication capacity, virus-host interaction, and pathogenesis will be crucial to the development of the best vaccine strategy.

Nonetheless, a comprehensive analysis of the atypical CHIKF symptoms due to the Brazilian outbreak from 2015 to 2019 is still lacking, since clinical data are scarce in the literature. Nevertheless, the impact of CHIKV in the Brazilian population could account for the introduction of a new pathogen into a naïve population with a higher probability to spread due to the highly populated urban areas and the high density of the mosquito vectors. However, the number of cases of CHIKV infection in Brazil, which were several orders of magnitude higher than in any other country of South America, was accompanied by the introduction of the ECSA strain, which substituted the Asian strain that was first introduced into the country. While in other regions of South America and in Central America, the Asian strain was responsible for the outbreaks. Thus, one cannot rule out the contribution of specific viral factors that allowed for the behavior of the epidemics in Brazil.

It is important to point out that the CHIKV introduction and epidemics in South America, and specifically in Brazil, occurred concomitantly with the epidemic of Zika virus, and the ongoing outbreaks of Dengue virus. Co-infections may promote the onset of serious illness, such as those with neurological symptoms. The number of co-infection cases still need to be fully addressed, but its impact on the clinical outcome of co-infected individual need to be anticipated.

Regarding viral-host interactions, it is clear that plenty of information on cellular processes is still a matter of debate, once depending on the cell type or animal model, some outcomes for the same question/issue can be quite contradictory. The deep comprehension of essential cellular processes that CHIKV can interfere with and alter to its own replication is a crucial task that researchers need to face and investigate. Thereby, results from new researches in the field of host-viral interaction could bring new strategies to combat this threat and to minimize the social, economic, and health burden, improving the life quality from the affected population, alleviating symptoms, avoiding some atypical complications, and interrupting viral persistence establishment.

## Author Contributions

MC, MS, IC, GS, and SC wrote the review and revised the figures. PAC and VF wrote the review. LC wrote and revised the review and revised the figures. All the authors contributed to the article and approved the submitted version.

## Conflict of Interest

The authors declare that the research was conducted in the absence of any commercial or financial relationships that could be construed as a potential conflict of interest.
